# Impact of lithotomy position on ureteral course and psoas muscle: a combined survey and radiological evaluation by the EAU Endourology & YAU Endourology working groups

**DOI:** 10.1007/s00345-026-06298-0

**Published:** 2026-02-24

**Authors:** Tarik Emre Sener, Naif Dinc Ulker, Turker Altuntas, Ersin Gokmen, Arman Tsaturyan, Amelia Pietropaolo, Begona Ballesta Martinez, Oriol Angerri, Andreas Skolarikos, Bhaskar Kumar Somani, Olivier Traxer

**Affiliations:** 1 School of Medicine, Department of Urology, Marmara University, Istanbul, Turkey; 2 Erebuni Medical Center, Yerevan, Armenia; 3 Department of Urology, University Hospital Southampton NHS Trust, Southampton, UK; 4 Hospital Universitario del Vinalopó, Elche, Alicante, Spain; 5 Fundació Puigvert, Department of Urology, Autonomous University of Barcelona, Barcelona, Spain; 6 Department of Urology, National and Kapodistrian University of Athens, Athens, Greece; 7 Department of Urology, Assistance-Publique Hôpitaux de Paris, Sorbonne University, Tenon Hospital, Paris, France; 8 Marmara Üniversitesi Pendik Eğitim ve Araştırma Hastanesi, Üroloji Anabilim Dalı, 4. Kat. Fevzi Çakmak Mahallesi, Pendik, Istanbul, Turkey

**Keywords:** Urolithiasis, Lithotomy, Psoas muscle, Minimally invasive stone surgeries

## Abstract

**Background and objective:**

To assess urologists’ perspectives on the impact of lithotomy position on the psoas muscle and ureteral course, and to radiologically evaluate these anatomical changes using a double J (DJ) stent as a marker.

**Methods:**

A prospective study was conducted between January and June 2025. An 11-item web-based survey was distributed to fellowship-trained endourologists to explore perceptions regarding lithotomy positioning during ureterorenoscopy. For radiological assessment, adults aged 18–65 who underwent DJ stent placement after ureteroscopy were analyzed using KUB X-rays obtained in supine and standard lithotomy positions. Distances between stent segments and lumbar vertebrae, as well as intersegmental angles, were measured and compared according to BMI.

**Key findings and limitations:**

Fifty-three urologists completed the survey. Most reported that psoas muscle length decreases (67.9%) and width increases (66%) in lithotomy position. The standard dorsal lithotomy position was preferred by 60.4%, and 62.3% believed positioning could affect ureteroscope passage at the iliac vessel crossover. Adjustable leg supports were routinely used by 84.9%. Increasing lithotomy level was not considered to significantly influence complication rates (69.8%), operative time (58.5%), or stone-free rates (62.3%). Among 22 radiologically evaluated patients (mean age 51.9 years, 64.6% male), no significant differences were found in stent–vertebra distances or segmental angles between positions, nor across BMI and gender groups.

**Conclusions and clinical implications:**

Although radiological analysis showed no measurable ureteral configuration changes, urologists perceive lithotomy positioning as potentially influential during endoscopic access. Further dynamic imaging and outcome-based studies are warranted to clarify its clinical significance.

## Introduction

Urolithiasis is one of the most common uropathologies with a prevalance rate between 2% and 20% [1, 2]. Management of urolithiasis includes a wide range of treatment modalities including surgical interventions as well as emergency decompression strategies tailored to patients’ stone burden, location and urgency status [3]. Although minimal invasive procedures such as retrograde intrarrenal surgery (RIRS) or percutaneous nephrolithotomy (PCNL) have been on the rise in the last decades, sometimes access to the stone might be challenging due to various conditions depending on patient anatomy, surgeon experience and technical equipments available.

Easy access to the upper urinary tract is one of the most important factors to decrease complications such as iatrogenic trauma or stricture. During minimally invasive stone surgeries such as ureteroscopy (URS), patients are commonly positioned in standard lithotomy position. In this set-up, the psoas muscle is expected to decrease in length and increase in width due to the flexion and abduction on the hip joint. However, we lack the exact anatomical evidence of the changes in the muscle morphology and dynamics and more importantly on the course of the ureter which lies on the muscle itself, especially on its proximal portion. Previous studies showed that adjusting patient position may change surgical outcomes [4–7], but none of these studies investigated the change of the course of the ureter due to leg movements during standard dorsal lithotomy position compared to supine position.

Although various studies have shown the higher success rates and lower complication rates with leg positioning adjustements, the underlying concept is interpreted differently among urologists [4, 8–10]. While not scientifically proven, opinions may vary about how the positioning of ureter and the psoas muscle alters when patients are shifted from supine to lithotomy position.

Accordingly, in this study, we aimed to explore endourologists’ perspectives on the relationship between lithotomy position, psoas muscle size and shape and course of the ureter. In addition, we sought to evaluate these potential changes radiologically, using a double J (DJ) stent as a marker of ureteral location when patients are shifted from supine to lithotomy position.

## Materials and methods

This study was a prospective clinical study designed at a tertiary care center. The study was conducted between January 2025 and June 2025. The study was approved by the Institutional Review Board (IRB) with the protocol number of Local Ethic Committee 09.2023.1064.

A structured, web-based survey consisting of eleven multiple-choice questions was distributed to members of YAU Endourology Working Party and EAU Endourology. All participants were fellowship-trained endourologists with substantial clinical experience (> 250 URS cases each). The aim of the survey was to explore the perceptions of endourology experts regarding lithotomy positioning, particularly its impact on anatomical relationships and procedural outcomes during ureterorenoscopy. The survey consisted of 11 multiple choice questions, allowing a single response selection.

Regarding the radiological evaluation, eligible patients aged 18–65 years who underwent ureteroscopy with subsequent DJ stent placement were included to minimize the potential confounding effects of age-related degenerative musculoskeletal changes on psoas morphology and the ureteral course. Exclusion criteria were: pregnant patients, patients who were unable to be positioned in lithotomy position due to skeletal deformities, patients with renal and ureteral abnormalities, patients with spinal diseases such as lordosis, kyphosis or scoliosis.

The patients included in the study were evaluated using KUB X-ray images obtained from both antero-posterior and sagittal aspects. The images were taken in both supine and standard lithotomy position. The standard lithotomy position was defined as abduction of legs to 45 degrees, with the hips flexed at an angle of 90 degrees.

**Fig. 1:**
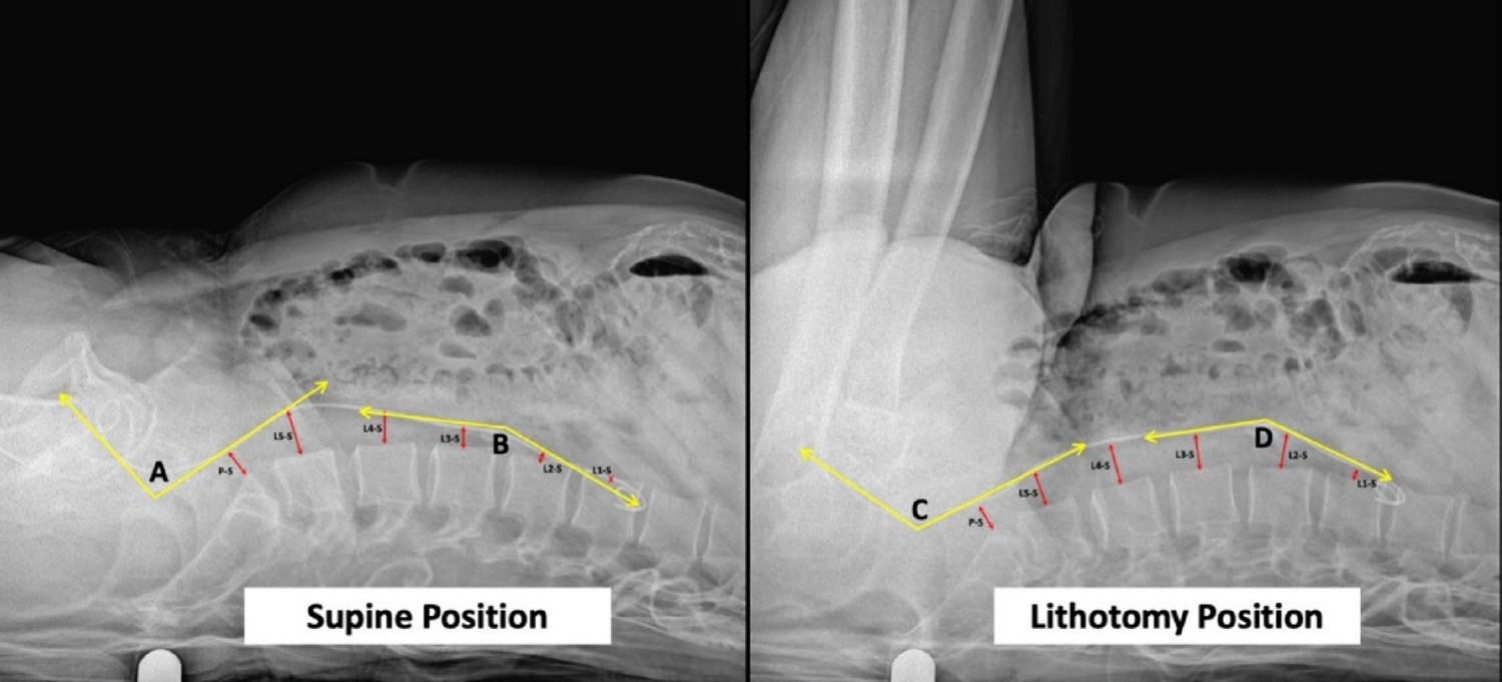
Measurement points. A: Angle between distal – mid part of the stent degree in supine position. B: Angle between proximal – mid part of the stent degree in supine position. C: Angle between distal – mid part of the stent degree in lithotomy position. D: Angle between proximal – mid part of the stent degree in lithotomy position. L: Lumbar Vertebrae. P: Promontorium. S: Ureteral Stent

All patients were positioned in the standard lithotomy position using adjustable leg supports. The ureteral stent, inserted prior to KUB X-ray, due to its opaque nature, was used to demonstrate the course of the ureter, and the changes of this course with the changes affecting the psoas muscle during lithotomy position compared to supine position. The distance of the ureteral stent to the corresponding lateral aspect of lumbar vertebrae bodies were measured for both positions. For the explanation of the segments of the stent; the stent was divided into three portions using predefined radiographic landmarks. The proximal segment was defined from the renal pelvis and upper coil to the first clear change in stent direction within the lumbar region (typically around L2–L3). The middle segment was defined as the subsequent lumbar portion extending to the point where the stent crosses the pelvic brim (identified radiographically by the entry into the bony pelvis). The distal segment was defined from the pelvic brim to the intravesical portion and lower coil. Angles were measured at the junctions between proximal–middle and middle–distal segments. The angles between each consecutive part were measured in both lithotomy and supine positions (Fig. 1). All distance and angle measurements on KUB X-rays were performed by a single experienced observer using a predefined and standardized measurement protocol to ensure methodological consistency. The changes in the aforementioned distances and the changes in angles were compared between lithotomy and supine positions.

Finally, a comparison of angle changes between positions was planned according to the patients’ body mass index (BMI). Participants with a BMI between 18.5 and 25 were classified as normal weight, those between 25 and 30 as overweight, and those above 30 as obese for the planned analysis.

### Statistical analyses

Statistical analyses were conducted using IBM SPSS 25.0. (Armonk, New York, United States). Due to non-normal data distribution (Kolmogorov-Smirnov test), non-parametric tests were applied. Numerical data were presented as median (min-max), categorical data as counts/percentages. The Chi-square test analyzed categorical data, while the Mann-Whitney and Kruskal-Wallis tests compared groups. A p-value < 0.05 was considered significant.

Descriptive statistics (frequencies and percentages) were used to summarize the results of the distributed survey.

## Results

### Survey results

The survey was sent to 55 urologists, with 53 completing it (96% response rate). Regarding the psoas muscle, 67.9% believed its length decreases and 66% thought its width increases in lithotomy; opinions on firmness were split (52.8% increase, 47.2% decrease). For ureteral position in dorsal lithotomy compared to supine, 34% reported ventral shift, 9.4% dorsal shift, 26.4% medial angulation at the iliac crossover, 17% lateral angulation at the iliac crossover, and 13.2% were unsure.

Most urologists (60.4%) preferred standard dorsal lithotomy (hip flexion 45–60°) for ureterorenoscopy. Regarding ureteroscope passage at the iliac crossover, 62.3% thought positioning possibly affects it, 18.9% definitely affects, and 18.8% were unsure or thought it does not. To overcome difficulties, 50.9% would switch to a flexible ureteroscope, 34% would place a ureteral stent and plan a second-look, and 15.1% would adjust patient position. Most (84.9%) routinely use adjustable leg supports.

**Fig. 2:**
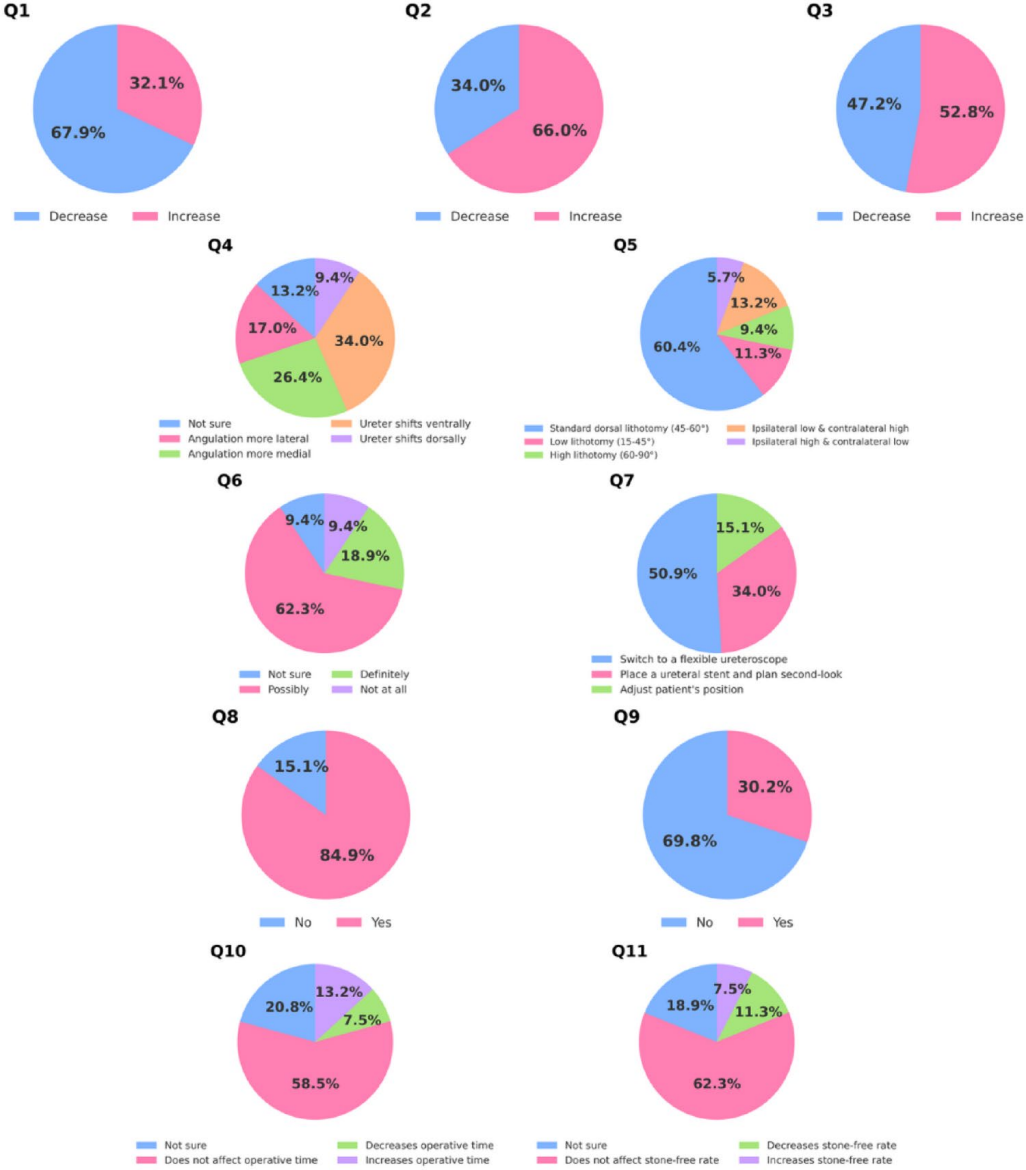
Survey response distribution

Concerning complications, 69.8% believed lithotomy level does not affect risk, while 30.2% thought it does. On operative time, 58.5% saw no effect, 13.2% an increase, 7.5% a decrease, and 20.8% were unsure. Similarly, 62.3% thought stone-free rates are unaffected by lithotomy level, 11.3% thought it decreases, 7.5% increases, and 18.9% were unsure (Table 1; Fig. 2).

**Table 1:** Survey results

Question		*n* (%)
1	When patients are positioned in lithotomy from supine position, which of the following changes occur in psoas muscle? [Length]	
	Decrease	36 (67.9)
	Increase	17 (32.1)
2	When patients are positioned in lithotomy from supine position, which of the following changes occur in psoas muscle? [Width]	
	Decrease	18 (34)
	Increase	35 (66)
3	When patients are positioned in lithotomy from supine position, which of the following changes occur in psoas muscle? [Firmness]	
	Decrease	25 (47.2)
	Increase	28 (52.8)
4	How does the position of the ureter change in the dorsal lithotomy position compared to the supine position?	
	Not sure	7 (13.2)
	The angulation of the ureter at the iliac vessel crossover becomes more lateral	9 (17)
	The angulation of the ureter at the iliac vessel crossover becomes more medial	14 (26.4)
	The ureter shifts ventrally in position	18 (34)
	The ureter shifts dorsally in position	5 (9.4)
5	What position do you prefer when performing ureterorenoscopy?	
	Standart dorsal lithotomy (hip flexion 45–60 degrees)	32 (60.4)
	Low lithotomy (hip flexion 15–45 degrees)	6 (11.3)
	High lithotomy (hip flexion 60–90 degrees)	5 (9.4)
	Ipsilateral low lithotomy and contralateral high lithotomy	7 (13.2)
	Ipsilateral high lithotomy and contralateral low lithotomy	3 (5.7)
6	Do you think patient positioning affects the passage of the ureterorenoscope at the level of the iliac vessel crossover?	
	Not Sure	5 (9.4)
	Possibly	33 (62.3)
	Definitely	10 (18.9)
	Not at all	5 (9.4)
7	What is your preferred strategy when encountering difficulty advancing the semirigid ureterorenoscope at the iliac vessel crossover?	
	Switch to a flexible ureteroscope	27 (50.9)
	Place a ureteral stent and plan a second-look procedure	18 (34)
	Adjust the patient’s position	8 (15.1)
8	Do you routinely use adjustable (moveable) leg supports during ureterorenoscopy?	
	No	8 (15.1)
	Yes	45 (84.9)
9	During ureterorenoscopy, do you think the level of lithotomy positioning affects the risk of complications such as ureteral avulsion or perforation?	
	No	37 (69.8)
	Yes	16 (30.2)
10	During ureterorenoscopy, how does an increase in the level of the lithotomy position affect operative time?	
	Not Sure	11 (20.8)
	It does not affect operative time	31 (58.5)
	It decreases operative time	4 (7.5)
	It increases operative time	7 (13.2)
11	During ureterorenoscopy, how does an increase in the level of the lithotomy position affect stone-free rate?	
	Not Sure	10 (18.9)
	It does not affect stone-free rate	33 (62.3)
	It decreases stone-free rate	6 (11.3)
	It increases stone-free rate	4 (7.5)

### Radiological evaluation results

Twenty two patients were included with a mean age of 51.9 +/- 6.8. Of patients included in the study, 14 (64.6%) were male and 8 were female (36.4%).

In the radiological analysis, the median distance between the ureteral stent and the lateral border of the lumbar vertebral bodies showed no statistically significant differences between the supine and lithotomy positions at any level. Specifically, the median stent–vertebra distances at the L1 level were 11.5 mm (range: 0–28) in the supine position and − 11.5 mm (range: −18 to 33) in the lithotomy position (*p* = 0.176). Similarly, at the L2, L3, L4, and L5 levels, the median distances were 9.5 mm vs. 13.5 mm (*p* = 0.217), 9.5 mm vs. 15 mm (*p* = 0.191), 14 mm vs. 15 mm (*p* = 0.224), and 14 mm vs. 15 mm (*p* = 0.866) for supine and lithotomy positions, respectively. The median distance at the level of the sacral promontory was 18 mm (range: 8–33) in the supine position and 18.5 mm (range: 5–32) in the lithotomy position (*p* = 0.399). With regard to ureteral configuration, the median angle between the distal and middle segments of the stent was 145.14° (range: 121–165) in the supine position and 146.92° (range: 126–162) in the lithotomy position (*p* = 0.49). Likewise, the median angle between the middle and proximal segments was 144.78° (range: 81–171) in the supine position and 145.21° (range: 81–172) in the lithotomy position, with no statistically significant difference between positions (*p* = 0.345) (Table 2).

**Table 2:** Comparison of stent position and configuration

	Supine	Lithotomy	*P* value
L1 – stent (mm)	11.5 (0–28)	-11.5 (-18-33)	0.176
L2 – stent (mm)	9.5 (-14-36)	13.5 (-22-44)	0.217
L3 – stent (mm)	9.5 (-11-43)	15 (-9-38)	0.191
L4 – stent (mm)	14 (-7-35)	15 (0–29)	0.224
L5 – stent (mm)	14 (-12-32)	15 (1–33)	0.866
Promontorium – stent (mm)	18 (8–33)	18.5 (5–32)	0.399
Angle between distal – mid part of the stent (degree)	145.14 (121–165)	146.92 (126–162)	0.49
Angle between mid – proximal part of the stent (degree)	144.78 (81–171)	145.21 (81–172)	0.345

All continuous variables are presented as median (minimum, maximum)

L: Lumbar vertebrae

Goniometric analysis of stent angulation changes in sagittal images showed that the median change at the distal–mid angle was − 9.5° (range: -32° to 20°) overall, while the median change at the mid–proximal angle was 0° (range: -17° to 7°). To explore whether sex-related anatomical differences might influence ureteral course, we compared angle changes between males (*n* = 14) and females (*n* = 8); no significant differences were observed for distal-mid or mid-proximal angle change (Mann–Whitney U test: distal U = 53.5, *p* = 0.864; proximal U = 35.0, *p* = 0.150). When stratified by weight groups, the distal–mid angle change was − 8.5° (-18° to 18°) in normal-weight patients, -8° (-32° to 11°) in high-weight patients, and − 14° (-30° to 20°) in obese patients. Corresponding mid–proximal angle changes were − 3.5° (-17° to 6°), 4° (-10° to 7°), and 0° (-12° to 2°), respectively. No statistically significant differences were observed between groups at either angle (*p* = 0.514 for normal-weight, *p* = 0.237 for high-weight, *p* = 0.225 for obese; overall *p* = 0.114) (Table 3).

**Table 3:** Goniometric analysis of stent angulation changes in sagittal images

	Distal – mid angle	Mid – proximal angle	*P* value
Overall (*n*:22) (degree)	-9.5 (-32-20)	0 (-17-7)	0.114
Subgroups			
Normal Weight (n:10) (degree)	-8.5 (-18-18)	-3.5 (-17-6)	0.514
High Weight (n:7) degree (degree)	-8 (-32-11)	4 (-10-7)	0.237
Obese (n:5) (degree)	-14 (-30-20)	0 (-12-2)	0.225

All continuous variables are presented as median (minimum, maximum)

## Discussion

Urolithiasis is a major global health problem due to its high prevalence, recurrent nature, and the associated morbidity it imposes [11]. Owing to advances in endourological surgery, ureteroscopy is one of the most commonly performed interventions for stone disease, with high success rates and low complication risks [12]. Despite these advantages, ureteroscopic access can sometimes be technically challenging, due to factors related to patient anatomy, surgeon experience, and positioning.

Recent experimental work has demonstrated that surgical positioning can also significantly alter intrarenal conditions during RIRS, with Trendelenburg position leading to higher intrarenal temperatures and prone split-leg producing the greatest intrarenal pressures, while the Galdakao-modified supine Valdivia showed the lowest pressures [7]. These data suggest that the choice of patient position may directly influence surgical safety and outcomes. Similarly, various positions such as putting the patients in T-tilt position (patient in a 45 degree Trendelenburg position and 45 degrees lateral tilt against the side of the stone) have been studied for getting better intraoperative vision, facilitating access to lower calyx, and facilitating clearance of stone fragments [5]. In another study by Lepine et al., the authors showed that reverse Trendelenburg position significantly reduced ureteral stone migration and necessity for flexible ureteroscopy whereas for renal stones Trendelenburg position facilitated migration of the stone to upper calyces and thereby increasing stone-free rates [4]. Although our study examined ureteral access changes related to lithotomy and psoas muscle configuration, and the referenced experimental work addressed intrarenal pressure and temperature in different setups, both highlight that patient positioning can critically influence surgical outcomes. In a recent study, Zeng et al. reported that adjustable boot stirrups enabled successful access in a challenging transplant kidney case complicated by a ureterovesical stricture and altered ureteral angle, illustrating how positioning aids can overcome anatomical barriers [6]. Likewise, our study on lithotomy and psoas-related ureteral course alterations supports the broader concept that patient positioning is not merely a technical detail but a key determinant of both access and safety.

There have always been attempts to optimize ureteral alignment and facilitate access in order to reduce ureteroscopic failure. One such approach has been the application of external lower-abdominal pressure to straighten the ureter over the iliac vessels [8], and in the same context, adjustments in leg positioning have been explored as another simple and low-cost strategy to achieve the same goal. In an earlier radiographic study, Angus and Webb provided important anatomical insight into how hip flexion alters the ureteral course. They demonstrated that the lower ureter has two distinct curves: a sacroiliac curve that straightens progressively with increasing hip flexion, and a vesical curve that remains unaffected by changes in position. The straightening effect results from anterior pelvic tilt and reduction in lumbar lordosis as the hips are flexed, thereby reducing angulation at the iliac bifurcation and facilitating endoscopic passage [9]. Their findings highlight that difficulties at the sacroiliac level are not primarily related to ureteric caliber, but rather to angulation, which can be mitigated by patient positioning. These radiological observations support the concept that positional changes modify the relationship between the ureter and surrounding structures such as the psoas muscle. In our study, radiological evaluation with ureteral stents did not reveal significant differences in ureteral angulation or relationship between supine and lithotomy positions, suggesting that while hip flexion can theoretically straighten the ureter at the sacroiliac level, these positional changes may not always translate into measurable alterations in ureteral configuration on imaging.

The effect of leg positioning on ureteral alignment during ureteroscopy has been clearly shown in previous work. In their randomized trial, Korkes et al. demonstrated that extending the ipsilateral leg in the lithotomy position reduced operative time compared to the standard position, particularly in men where anatomical constraints are more evident [10]. The authors suggested that this simple maneuver helps to reduce angulation and kinking of the ureter, making endoscopic passage easier and improving ergonomics. Editorial comments on this study highlighted the practical value of such a low-cost adjustment, while also noting the importance of surgical experience and other anatomic factors, such as prostate size, that may influence ureteral access [10]. These findings parallel our investigation into the relationship between the psoas muscle and ureteral positioning when patients are shifted from supine to lithotomy. Just as leg extension alters the ureteral course, changes in the psoas–ureter axis during positional shifts may also impact surgical access and difficulty, emphasizing the relevance of patient positioning in endourology.

In our study, despite not finding measurable radiological differences in ureteral alignment between the two positions, these clinical observations remain relevant, as they highlight that even subtle anatomical or positional influences, undetectable by static imaging, may still play a role in operative ergonomics and surgical performance.

In this study, we also combined expert perspectives with radiological evaluation to investigate the effects of the lithotomy position on the psoas muscle and the ureter. Using a web-based survey, we aimed to evaluate the opinions of expert endourologists on the potential anatomical changes in the psoas muscle and ureteral course, as well as their perceptions of the success and complication rates, when patients are shifted from supine to lithotomy position. Additionally, by using a DJ stent as a radiological marker, we aimed to provide the understanding into how patient positioning may alter ureteral course.

This survey provides insight into the clinical perceptions of experienced endourologists regarding the anatomical and procedural consequences of lithotomy positioning. Despite the absence of a universal agreement on specific changes, most respondents acknowledged that lithotomy alters the course of the ureter and characteristics of the psoas muscle, particularly shortening and increased firmness. These perceptions align with the concept that retroperitoneal tension and muscular dynamics may affect ureteral mobility and angulation.

The overwhelming preference for standard lithotomy with moderate hip flexion suggests an effort to balance access and patient safety. The majority of respondents not only believe positioning affects the ease of advancing a ureterorenoscope, especially at the iliac crossover — but also actively adjust patient position as their primary corrective maneuver.

The routine use of adjustable leg supports by over 80% of respondents reinforces the importance of intraoperative flexibility. Interestingly, while most agreed that lithotomy level may affect complication rates, particularly avulsion or perforation, fewer believed it has a significant impact on stone-free rates or operative time. This suggests that while safety concerns are high, procedural success may be perceived as more resistant to positional variation.

Although our radiological analysis did not show a significant change in the ureteral course between supine and lithotomy positions, 62.3% of expert endourologists believe that patient positioning affects ureteroscope passage. We believe that this discrepancy may be influenced by factors not captured by static imaging. For example, dynamic ureteral mobility, peristaltic activity, and subtle tissue compliance differences may facilitate or hinder scope advancement. In addition, operator experience and subjective assessments of resistance during ureteroscope insertion may affect the perceived ease of passage, even when measurable angle or distance changes are minimal. These findings suggest that, although imaging provides objective anatomical data, ureteroscope manipulation may still be influenced by functional and procedural nuances.

### Limitations

This study has limitations. The radiological evaluation was performed in a relatively small cohort, which may have reduced the power to detect subtle positional differences, and the small numbers within the BMI subgroups also make those subgroup comparisons less reliable. We assessed only static imaging in the supine and lithotomy positions, so we could not capture dynamic changes that may occur during actual ureteroscope manipulation; real-time assessment (such as fluoroscopy during leg movement) might better demonstrate transient angulation and subtle positional shifts that static KUB imaging can miss. In addition, using a DJ stent as a marker for ureteral course may introduce some bias, since the stent’s shape and rigidity can partially straighten the ureter and potentially mask its mobility. Another limitation is the lack of standardized cross-sectional imaging (CT/MRI), as routine postoperative CT or MRI is not recommended by current guidelines; therefore, we could not perform quantitative psoas morphometry or explore its relationship with positional changes. Finally, these findings may not fully generalize to all patient groups, where age-related musculoskeletal changes and pelvic/spinal anatomical changes may influence ureteral course and positional dynamics differently.

## Conclusion

This study demonstrates for the first time that urologists, including expert endourologists, have differing perspectives on ureteral course changes, which may impact surgical outcomes. Survey results indicate that lithotomy positioning, particularly hip flexion, is believed to influence the psoas muscle, ureter, and procedural dynamics during ureterorenoscopy. While opinions on specific anatomical changes varied, there was consensus that positioning affects scope advancement and may contribute to complications. Radiological evaluations suggest that lithotomy position reduces ureteral steepness by altering its anterior-posterior course, though changes were not statistically significant. Overall, these findings highlight that patient positioning may affect ergonomics and perceived ease of ureteral access, warranting further dynamic and outcome-based studies.

## Data Availability

No datasets were generated or analysed during the current study.
